# Medical advertising on social media

**DOI:** 10.1016/j.bjorl.2022.05.004

**Published:** 2022-05-24

**Authors:** Gustavo Polacow Korn, Vania Rosa Moraes, Virgilio Prado

**Affiliations:** a Universidade Federal de São Paulo, São Paulo, SP, Brazil; b Former President of the Academia Brasileira de Laringologia e Voz (ABLV), Brazil; c Associação Brasileira de Otorrinolaringologia e Cirurgia Cérvico-Facial (ABORL-CCF), Departamento Jurídico, São Paulo, SP, Brazil; d Associação Brasileira de Otorrinolaringologia e Cirurgia Cérvico-Facial (ABORL-CCF), Comitê de Defesa Profissional e da Comissão de Telemedicina, São Paulo, SP, Brazil

Social networks play an important role in disseminating relevant information and content to the public. Its use for medical advertising is a subject that raises many doubts, controversies, and concerns. Clarification about these issues is important for the physician to disseminate ethical, educational, informative content and to somehow help their patients and society in general regarding health-related issues.

The physician is free to maintain accounts on social networks and create content related to their work. However, there are specific rules for medical advertising that must be followed.^1^ The decorum of the profession, respect for medical colleagues and the preservation of the confidentiality of the patient’s image and identity are essential points of the medical advertising rules and the reason why several styles of posting are prohibited. In compliance with the rules, the physician must be careful so that their posts on social networks do not include contents with sensationalist, self-promotion, and unfair competition characteristics.

For the reasons highlighted above, pictures of patients before and after treatments should not be displayed, nor should any other type of picture or text indicating or implying good results.^2^ Pictures with celebrities or easily recognizable personalities undergoing medical treatment are also prohibited, which forbids hiring or partnering with digital influencers. Unfortunately, all of the above situations are frequently observed in medical social networks, despite being considered ethical infractions that may lead the physician to respond to ethical-professional lawsuits to the Medical Council.^3^

The physician is also free to grant interviews with medical and health content in the most diverse media. During these interviews, physicians must be properly identified with their full name, CRM (Regional Council of Medicine) number, specialty and Specialty Qualification Record (RQE, *Registro de Qualificação de Especialidade*) number. Interviews should be seen as an oportunity to inform the population about topics related to medicine and health and not as a chance for publicity. In addition to respecting all the previously mentioned ethical rules, the physician must not use these interviews to disclose the address and telephone number of their office or clinic, specialty or area of ​​activity that they cannot prove through the RQE number, publicizing consultation and procedure fees or moreover, offering services as a gift or gratuity of any kind.

Another important point that must be clarified, is that the social network is not the appropriate environment for medical care and should not replace the doctor-patient relationship, whether in person or by telemedicine. We highlight that the use of question-and-answer tools has been increasing on social networks and many doctors have responded by giving complete diagnoses and prescriptions, practices that are prohibited by the ethical standards and that can be used as evidence in eventual administrative and judicial lawsuits.

The physician's or clinic homepage continues to be a relevant dissemination tool, especially due to their power of visibility in internet search tools. The page can include clinical staff information, such as training and curriculum data, as well as the clinic contact data, such as its telephone number and address, both physical and in social media.^4^ The medical technical director’s name, CRM and EQR must be obligatorily displayed. The Homepage, as it is a broader room, can contain more complete data, such as the addressed pathologies and types of treatments performed by the clinic. One should always be careful to avoid self-promotion, sensationalism, and unfair competition.

The ABORL-CCF Legal Department and the Professional Defense Committee are at their member's disposal to answer any doubts regarding medical advertising. Ideally, one should avoid practices that can be unethical, leading to unfair competition with profession and specialty colleagues being subject of a complaint to the Regional Council of Medicine, which can lead to the opening of an inquiry and to ethical-professional lawsuits to the detriment of the physician.^5^

## Conflicts of interest

The authors declare no conflicts of interest.
